# Higher peripheral blood mitochondrial DNA copy number and relative telomere length in under 48 years Indonesian breast cancer patients

**DOI:** 10.1186/s13104-024-06783-y

**Published:** 2024-04-28

**Authors:** Prisca C. Limardi, Sonar Soni Panigoro, Nurjati Chairani Siregar, Noorwati Sutandyo, Fiastuti Witjaksono, Lidwina Priliani, Sukma Oktavianthi, Safarina G. Malik

**Affiliations:** 1 Master’s Programme in Biomedical Sciences, Faculty of Medicine, Universitas Indonesia, Jakarta, Indonesia; 2 Genome Diversity and Diseases Laboratory, Eijkman Institute for Molecular Biology, Jakarta, Indonesia; 3 Genome Diversity and Diseases Division, Mochtar Riady Institute for Nanotechnology, Jl. Boulevard Jenderal Sudirman 1688, Lippo Karawaci, Tangerang, Banten 15811 Indonesia; 4 Department of Surgical Oncology, Dr. Cipto Mangunkusumo Hospital-Faculty of Medicine, Universitas Indonesia, Jakarta, Indonesia; 5 Department of Anatomical Pathology, Dr. Cipto Mangunkusumo Hospital-Faculty of Medicine, Universitas Indonesia, Jakarta, Indonesia; 6 Department of Hematology and Medical Oncology, Dharmais Hospital National Cancer Center, Jakarta, Indonesia; 7 Department of Nutrition, Dr. Cipto Mangunkusumo Hospital-Faculty of Medicine, Universitas Indonesia, Jakarta, Indonesia

**Keywords:** Breast cancer, mtDNA-CN, RTL, Oxidative stress, qPCR

## Abstract

**Objective:**

Breast cancer is the leading cause of cancer incidence and mortality among Indonesian women. A comprehensive investigation is required to enhance the early detection of this disease. Mitochondrial DNA copy number (mtDNA-CN) and relative telomere length (RTL) have been proposed as potential biomarkers for several cancer risks, as they are linked through oxidative stress mechanisms. We conducted a case–control study to examine peripheral blood mtDNA-CN and RTL patterns in Indonesian breast cancer patients (n = 175) and healthy individuals (n = 181). The relative ratios of mtDNA-CN and RTL were determined using quantitative real-time PCR (qPCR).

**Results:**

Median values of mtDNA-CN and RTL were 1.62 and 0.70 in healthy subjects and 1.79 and 0.73 in breast cancer patients, respectively. We found a positive association between peripheral blood mtDNA-CN and RTL (*p* < 0.001). In under 48 years old breast cancer patients, higher peripheral blood mtDNA-CN (mtDNA-CN ≥ 1.73 (median), *p* = 0.009) and RTL (continuous variable, *p* = 0.010) were observed, compared to the corresponding healthy subjects. We also found a significantly higher ‘High-High’ pattern of mtDNA-CN and RTL in breast cancer patients under 48 years old (*p* = 0.011). Our findings suggest that peripheral blood mtDNA-CN and RTL could serve as additional minimally invasive biomarkers for breast cancer risk evaluation.

**Supplementary Information:**

The online version contains supplementary material available at 10.1186/s13104-024-06783-y.

## Introduction

Breast cancer has been the leading cause of cancer incidence globally in 2020 and continues to be the primary cause of cancer-related deaths among women [1, 2]. It is also the most common type of cancer in Indonesia, with a higher age-standardised death rate (15.3 per 100,000) [3] than the global mortality rate (13.6 per 100,000) in 2020 [4], indicating a relatively lower survival rate for breast cancer patient in Indonesia.

The search for non-invasive biomarkers for breast cancer screening remains a challenging process. One of these efforts involves exploring the potential utilisation of the peripheral blood mitochondrial DNA copy number (mtDNA-CN) and relative telomere length (RTL) [5–7].

The relationship between mitochondria and telomeres has been studied extensively, particularly in biological ageing. They are intertwined through the telomere-p53-PGC–1α-mitochondria axis and are intricately linked to oxidative stress [8]. Their functionality is commonly estimated by measuring the mtDNA-CN and RTL. Nevertheless, studies that simultaneously incorporating both biomarkers, particularly in association with breast cancer, remain limited. Independent studies with peripheral blood mtDNA-CN [6, 9–13] and RTL [5, 14–20] have also reported inconsistent findings. However, a prospective study by Campa et al. found a positive association between high peripheral blood mtDNA-CN and high RTL, along with an increased risk of breast cancer [7], suggesting that peripheral blood mtDNA-CN and RTL could be used as minimally invasive biomarkers for breast cancer risk evaluation.

Our study aimed to investigate the differences in peripheral blood mtDNA-CN and RTL between breast cancer patients and healthy subjects in Indonesia. To the best of our knowledge, such a study has not been conducted in Indonesia before. We hypothesise that there are significant differences in both biomarkers between breast cancer patients and healthy subjects, which can potentially be utilised as additional minimally invasive biomarkers for breast cancer risk evaluation in Indonesia.

## Methods

### Study design and participants

This retrospective case–control study was initially conducted between 2019 and 2020, following specific inclusion and exclusion criteria [21]. The case subjects are females diagnosed with breast cancer based on histopathology and immunohistochemistry assay, aged 19 years or older, who have not undergone any cancer therapies. The control subjects are healthy disease-free females, aged 19 years or older, without any history of cancer and chronic illnesses. Following the incorporation of incomplete data as additional exclusion criteria, this study further investigated 175 breast cancer patients and 181 healthy subjects from six public referral hospitals in Indonesia (Additional file 1: Fig. S1). Approval from the Ethical Committee of Health Research at the Faculty of Medicine, Universitas Indonesia, Rumah Sakit Cipto Mangunkusumo, Jakarta, Indonesia, was obtained under reference number 450/UN2. F1/ETIK/2018.

### Clinical samples and data measurements

We analysed 356 archived peripheral blood samples stored at −70 °C. Demographic data were obtained using a self-administered questionnaire, including age, menarche age, menopause, childbirth history, breastfeeding, hormonal contraceptive use, smoking status, and alcohol consumption. Body mass index (BMI) was calculated by dividing weight (kg) by height squared (m^2^). Serum lipid concentrations were measured after 12 h of overnight fasting, including triglycerides (TG), high-density lipoprotein-cholesterol (HDL-C), low-density lipoprotein-cholesterol (LDL-C), and total cholesterol (TC). We also calculated the triglyceride glucose (TyG) index using the following equation: Ln[fasting TG (mg/dL) x FPG (mg/dL)/2] [22].

### Measurement of peripheral blood mtDNA-CN and RTL

Total DNA was extracted from archived peripheral blood samples using the salting-out methods. Blood samples with a total volume of 3 mL were extracted by employing Gentra® Puregene® Blood Kit (Qiagen, Hilden, Germany). Meanwhile, samples with a total volume of 1 mL were extracted using Geneius™ Micro gDNA Extraction Kit (Geneaid Biotech Ltd., New Taipei City, Taiwan), following both manufacturers’ instructions. Despite different extraction kits, no significant differences were found in mtDNA-CN and RTL measurement results (Additional file 1: Table S1).

Each extracted DNA sample was diluted with nuclease-free water (Ambion, Texas, USA) to a concentration of 5 ng/μL and used as a quantitative real-time PCR (qPCR) template. The reactions were carried out using *Power* SYBR^™^ Green PCR Master Mix (Applied Biosystems, California, USA) on a 7500 Real-Time PCR System (Applied Biosystems, California, USA). Each sample was evaluated in duplicate. The mtDNA-CN [23] and RTL [24] were measured by calculating their relative ratios to Beta-2-microglobulin (*B2M*), the single-copy nuclear-encoded reference gene. This calculation was based on the efficiency-corrected method implemented in the “qpcR” package [25]. A list of the primer pairs is provided in Additional file 1: Table S2.

### Statistical analyses

Data analysis was performed using R version 4.1.2 (www.r-project.org) with R Studio (version 2021.9.2.382; www.rstudio.com). The normality of continuous variables was evaluated using the Shapiro–Wilk test. Medians (interquartile ranges) were reported for non-normally distributed variables (age, BMI, TG, HDL-C, LDL-C, TC, FPG, TyG index) and evaluated using the Wilcoxon-Mann Whitney U test. Categorical variables (alcohol consumption, smoking, menarche age, menopause, childbirth history, breastfeeding, hormonal contraceptive use) were evaluated using Pearson’s chi-square test and reported as the number of samples (percentage). Since the relative ratios of mtDNA-CN and RTL were not normally distributed even after being transformed into log_e_, their associations were estimated using rank-based linear regression models using the “Rfit” package [26]. Odds ratios (ORs) with 95% confidence intervals (CIs) were calculated using the likelihood ratio test, adjusted for potential confounders. Variable selections incorporated in the model were carried out using stepwise multiple regression implemented in the “MASS” package [27]. Confounders added to the models were age, BMI, HDL-C, LDL-C, TC, TyG index, alcohol consumption, and smoking status. Analyses were carried out on all subjects and sub-groups based on age (under 48 years and above 48 years subgroups). The cut-off age of 48 years was determined by the ‘SpEqualSe’ method in the “OptimalCutpoints” package, based on the equality of specificity and sensitivity values [28]. The effect size and power calculation were done using the “pwr” package [29]. Significance was indicated by a *p*-value of < 0.025, following Bonferroni correction.

## Results

### Characteristics of study participants

The characteristics of the study participants are shown in Additional file 1: Table S3. Compared to the healthy subjects, breast cancer patients had significantly higher median age (median value, healthy group vs. breast cancer group, 45 years vs. 48 years, *p* = 0.003) and lower BMI (*p* = 0.003). Several clinical characteristics also showed significant differences (all *p* < 0.050). Breast cancer patients had a higher TG, lower HDL-C, higher FPG, and TyG index compared to healthy subjects. The prevalences of alcohol consumption and smoking were significantly higher in breast cancer patients. Those who developed breast cancer had a notably higher frequency of childbirth history, breastfeeding for less than 12 months, and hormonal contraceptive use.

### The positive association between peripheral blood mtDNA-CN and RTL

**Fig. 1:**
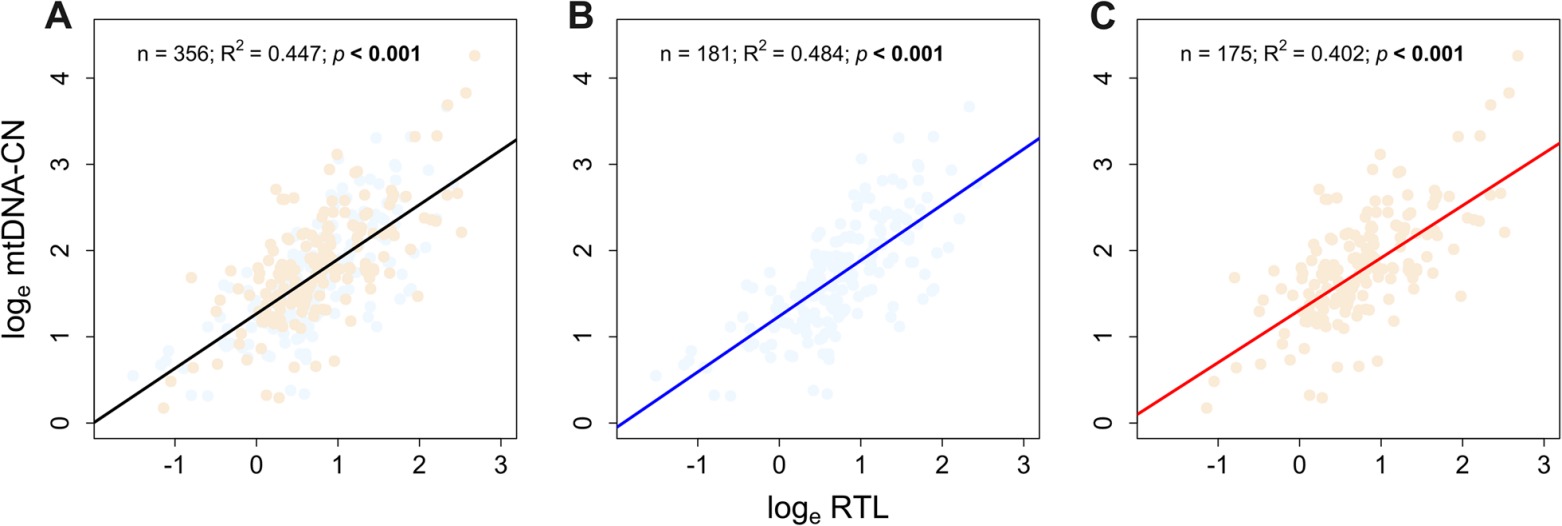
The positive association between peripheral blood mtDNA-CN and RTL. MtDNA-CN and RTL were positively associated with one another in **A** all samples, **B** healthy subjects, and **C** breast cancer patients

We found a consistent positive association between peripheral blood mtDNA-CN and RTL in all subjects and healthy and breast cancer groups. The association was found noteworthy (*p* < 0.001; *R*^*2*^ > 0.4) in each group, as shown in Fig. 1.

### Comparison of peripheral blood mtDNA-CN and RTL between breast cancer patients and healthy subjects

The univariate comparison of peripheral blood mtDNA-CN and RTL between breast cancer patients and healthy subjects is presented in Additional file 1: Fig. S2. The peripheral blood mtDNA-CN was significantly higher in the breast cancer patients compared to the healthy subjects (median value, healthy subjects *vs.* breast cancer patients, 1.62 vs. 1.79; *p* = 0.038). However, no significant differences in RTL were found between the two groups.

Additional analyses were performed on age subgroups, specifically those under and over 48 years old. Additional file 1: Fig. S3 displays the results of a univariate comparison between mtDNA-CN and RTL levels in peripheral blood samples for healthy individuals and breast cancer patients in each age subgroup (under and above 48 years). Our findings indicated that mtDNA-CN (*p* = 0.059) and RTL (*p* = 0.008) tended to be higher in healthy subjects aged 48 years and above. In contrast, RTL levels (*p* = 0.046) tended to be lower in breast cancer patients aged above 48 years.

**Fig. 2:**
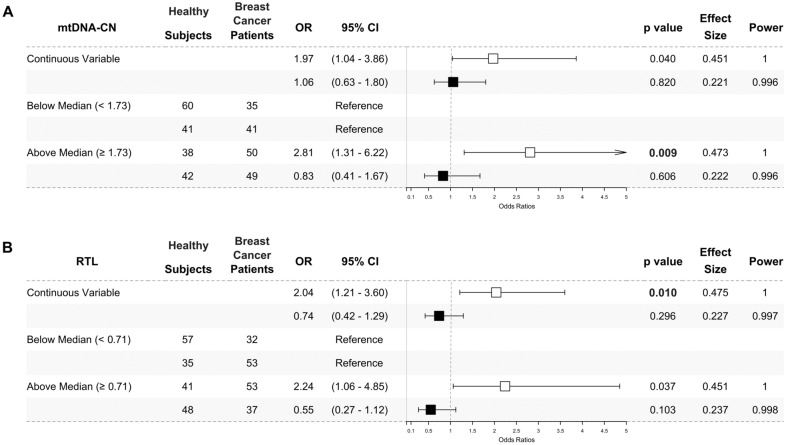
Higher peripheral blood mtDNA-CN (**A**) and RTL (**B**) in under 48 years breast cancer patients. The odds ratios of multivariate analysis for mtDNA-CN (**A**) and RTL (**B**) were presented as forest plots. The under 48 years subgroup was presented as white square (□). The above 48 years subgroup was presented as black square (■). The mtDNA-CN and RTL were analysed as a continuous variable and classified by the median value. Peripheral blood mtDNA-CN were significantly higher in under 48 years (□) breast cancer patients when analysed using the median cut-off (1.73) and nominally significant when analysed as a continuous variable. Meanwhile, peripheral blood RTL was significantly higher in under 48 years (□) breast cancer patients when being analysed as a continuous variable and nominally significant when being analysed using the median cut-off (0.71). No significant result was found in the above 48 years (■) subgroup. The likelihood ratio test was used to calculate odds ratios (ORs) with 95% confidence intervals (95% CIs) for association studies. Potential confounders added to the models were age, BMI, HDL-C, LDL-C, TC, TyG index, alcohol consumption, and smoking status. Significance was indicated by a *p*-value of < 0.025, following Bonferroni correction

We also conducted multivariate analyses in the age subgroups and presented the results as odds ratios. Breast cancer patients in the under 48 years subgroup had a significantly higher mtDNA-CN when analysed with classified median value (mtDNA-CN ≥ 1.73 (median), OR 2.81, 95% CI 1.31–6.22, *p* = 0.009, power = 1), and showed nominal significance when analysed as a continuous variable (OR 1.97, 95% CI 1.04–3.86, *p* = 0.040, power = 1) (Fig. 2A).

RTL was also higher in under 48 years breast cancer patients when analysed as a continuous variable (OR 2.04, 95% CI 1.21–3.60, *p* = 0.010, power = 1) and showed a nominal significance when analysed with classified median value (RTL ≥ 0.71 (median), OR 2.24, 95% CI 1.06–4.85, *p* = 0.037, power = 1) (Fig. 2B) as compared to the corresponding healthy subjects. Meanwhile, no significant odds ratios were found in the above 48-year subgroup.

**Fig. 3:**
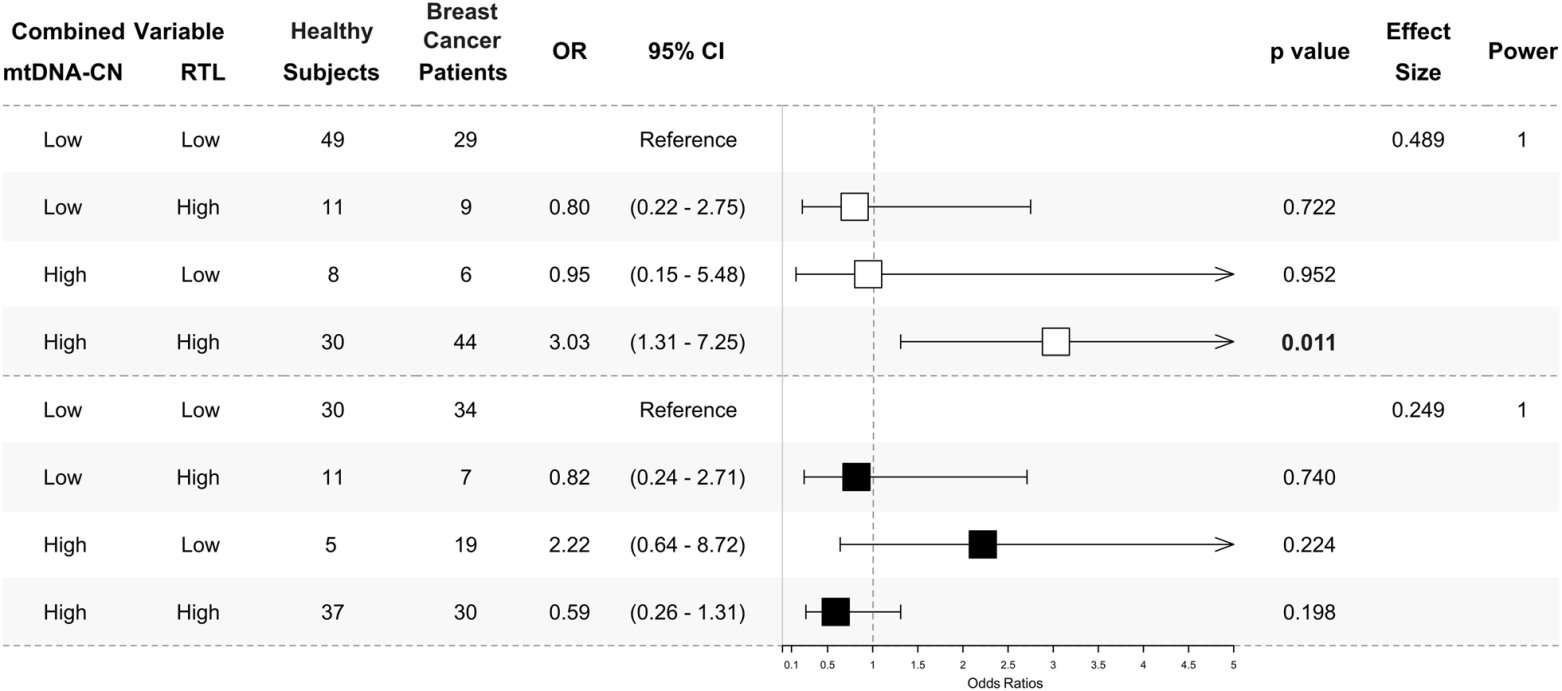
‘High-High’ pattern of peripheral blood mtDNA-CN and RTL in under 48 years breast cancer patients. The odds ratios of multivariate analysis for the mtDNA-CN and RTL combination pattern were presented as a forest plot. The under 48 years subgroup was presented as white square (□). The above 48 years subgroup was presented as black square (■). The combination was determined by the median value of each marker. The median cut-off is 1.73 for mtDNA-CN and 0.71 for RTL. A value below the median ( <) was classified as ‘Low’ and above the median ( ≥) as ‘High’. The ‘Low-Low’ combination was used as a reference. A ‘High-High’ combination was significantly found to be higher in under 48 years (□) breast cancer patients. No significant result was found in the above 48 years (■) subgroup. The multinomial regression test was used to calculate odds ratios (ORs) with 95% confidence intervals (95% CIs) for association studies. Potential confounders added to the models were age, BMI, HDL-C, LDL-C, TC, TyG index, alcohol consumption, and smoking status. Significance was indicated by a *p*-value of < 0.025, following Bonferroni correction

We analysed the combination patterns of mtDNA-CN and RTL in peripheral blood using their median values. Values below the median were categorised as ‘Low’, while those above were considered 'High'. The median values for mtDNA-CN and RTL are 1.73 and 0.71, respectively. Consistent with individual analysis, we identified a significantly higher ‘High-High’ pattern of mtDNA-CN and RTL in breast cancer patients under 48 years old compared to healthy subjects of the same age group (OR 3.03, 95% CI 1.31–7.25, *p* = 0.011, power = 1). However, there were no significant differences in ‘Low–High’ and ‘High-Low’ patterns in both subgroups under and above 48 years old (*p* > 0.050, power = 1), as depicted in Fig. 3.

## Discussion

Breast cancer is a complex disease influenced by numerous factors. The development and presence of the disease contribute to systemic changes. These alterations can be observed in circulating blood, leading breast cancer to be recognised as a systemic disease [30]. Furthermore, in addition to its minimally invasive collection methods, blood circulates throughout the body and contains information regarding systemic status, e.g., the body’s response to malignancies [31]. This study observed distinct peripheral blood mtDNA-CN and RTL patterns between Indonesian breast cancer patients and healthy subjects, particularly in the under 48 years subgroup.

The mtDNA-CN and RTL are commonly associated with the ageing process, and numerous studies have highlighted an inverse relationship between these markers and age [32, 33]. Both biomarkers exhibit dynamic regulation in response to environmental changes, mainly with antioxidant-oxidant imbalance. The lack of histone and less-adequate repair mechanism in mtDNA [34] and high-guanine residue in telomeres makes them susceptible to oxidative stress exposure [35]. Following previous studies in healthy adults [36, 37], pregnant women [38], gastric cancer tissues [39], and breast cancer patients [40], our study also found a positive association between peripheral blood mtDNA-CN and RTL, indicating their significant interrelationship.

Our multivariate analysis revealed that breast cancer patients, particularly those under 48 years, exhibited a higher peripheral blood mtDNA-CN and RTL than healthy subjects. An increase in peripheral blood mtDNA-CN is potentially due to the activation of mitochondrial compensatory mechanism to maintain mitochondrial function in response to prolonged oxidative stress exposure during disease progression [41–44]. The dynamic regulation of blood telomere length occurs during hematopoiesis in the bone marrow. In the case of chronic diseases like breast cancer, prolonged exposure to oxidative stress may stimulate telomere lengthening as compensation for telomere loss in hematopoietic cells, which can later be observed in the peripheral blood cells [45]. Our finding also revealed that the ‘High-High’ combined pattern of both biomarkers was predominant in breast cancer patients under 48 years, which is consistent with previous research [7].

In separate and combined mtDNA-CN and RTL analyses, the subgroup of individuals under 48 years old consistently showed statistically significant results. This observation can be attributed to several factors. Firstly, it is worth noting that 48 years corresponds to the average age of menopause for Indonesian women [46–48]. Secondly, younger individuals may possess a better capacity for the compensatory mechanism of mitochondria and telomeres. The differences are more pronounced when comparing a relatively younger case–control subgroup [49, 50].

The regulation of mtDNA-CN and RTL can be affected by the level and duration of exposure to oxidative stress [51, 52]. Studies have shown that breast cancer patients typically exhibit higher levels of oxidative stress compared to healthy individuals [53]. Various risk factors for breast cancer can contribute to the development of oxidative stress during breast carcinogenesis. In this study, we identified several significant differences in specific clinical parameters (TG, HDL-C, FPG, TyG index) and lifestyle factors (alcohol consumption, smoking, breastfeeding duration, hormonal contraceptive use) between the breast cancer and healthy groups (Additional file 1: Table S3). These variables are known to increase the risk of breast cancer. Alcohol consumption [54], smoking [55], and lifestyle changes that lead to alterations in serum lipid concentrations [56, 57] have been linked to increased oxidative stress levels through various mechanisms.

On the other hand, cancer cells also produce ROS due to their high metabolic rate, accumulation of genetic alterations, relative hypoxia, and persistent inflammation [58]. Therefore, the altered level of oxidative stress in breast cancer patients might have resulted from either the presence of risk factors during carcinogenesis or the production of ROS by cancer cells. Nevertheless, determining the underlying mechanisms from a case–control study perspective can be pretty challenging.

## Limitations

It is important to note that this study has some limitations. Firstly, the study cannot be generalised to other populations. Secondly, we did not measure any oxidative stress markers, which could provide valuable insights into the underlying mechanisms of mtDNA-CN and RTL regulations in breast cancer. Thirdly, the retrospective case–control study design cannot explain the direction of causation. Hence, future prospective studies enrolling other populations are warranted.

## Conclusion

In summary, we have identified distinctive peripheral blood mtDNA-CN and RTL characteristics in under 48 years breast cancer patients. This pilot case–control study for Indonesian breast cancer patients has highlighted a potential utilisation of both biomarkers as additional minimally invasive tools for enhancing early breast cancer risk evaluation.

### Supplementary Information

**Additional file 1.** Figure S1. Flow diagram of the healthy subjects and breast cancer (BC) patients' enrolment Table S1. Comparison of mtDNA-CN and RTL between extraction methods Table S2. List of primer pairs Table S3. Characteristics of study participants Figure S2. Univariate comparison of peripheral blood mtDNA-CN and RTL between healthy subjects and breast cancer patients Figure S3. Univariate comparison of peripheral blood mtDNA-CN and RTL between under and above 48 years subgroup in healthy subjects and breast cancer patients.

## Data Availability

The dataset supporting the conclusion of this article is available in the Mendeley Data repository, doi: https://dx.doi.org/10.17632/v7cz97s659.1

## Abbreviations

mtDNA-CN: Mitochondrial DNA copy number
RTL: Relative telomere length
qPCR: Quantitative polymerase chain reaction
OR: Odds ratio
95% CI: 95% Confidence interval
ROS: Reactive oxygen species
TG: Triglycerides
TC: Total cholesterol
FPG: Fasting plasma glucose
TyG index: Triglyceride and glucose index
HDL-C: High density lipoprotein-cholesterol
LDL-C: Low density lipoprotein-cholesterol
PGC-1α: Peroxisome proliferator-activated receptor-gamma coactivator-1 alpha
B2M: Beta-2 microglobulin
BMI: Body mass index
