# Supplementary material for: Higher peripheral blood mitochondrial DNA copy number and relative telomere length in under 48 years Indonesian breast cancer patients
Source: BMC Res Notes. 2024 Apr 28;17:120. doi: 10.1186/s13104-024-06783-y (PMC11057172; doi:10.1186/s13104-024-06783-y)
Supplement: Supplementary file 1 — Additional file 1. Figure S1. Flow diagram of the healthy subjects and breast cancer (BC) patients' enrolment Table S1. Comparison of mtDNA-CN and RTL between extraction methods Table S2. List of primer pairs Table S3. Characteristics of study participants Figure S2. Univariate comparison of peripheral blood mtDNA-CN and RTL between healthy subjects and breast cancer patients Figure S3. Univariate comparison of peripheral blood mtDNA-CN and RTL between under and above 48 years subgroup in healthy subjects and breast cancer patients. [file 13104_2024_6783_MOESM1_ESM.zip › Additional file/rev-Supplementary Figure 2.docx]

**
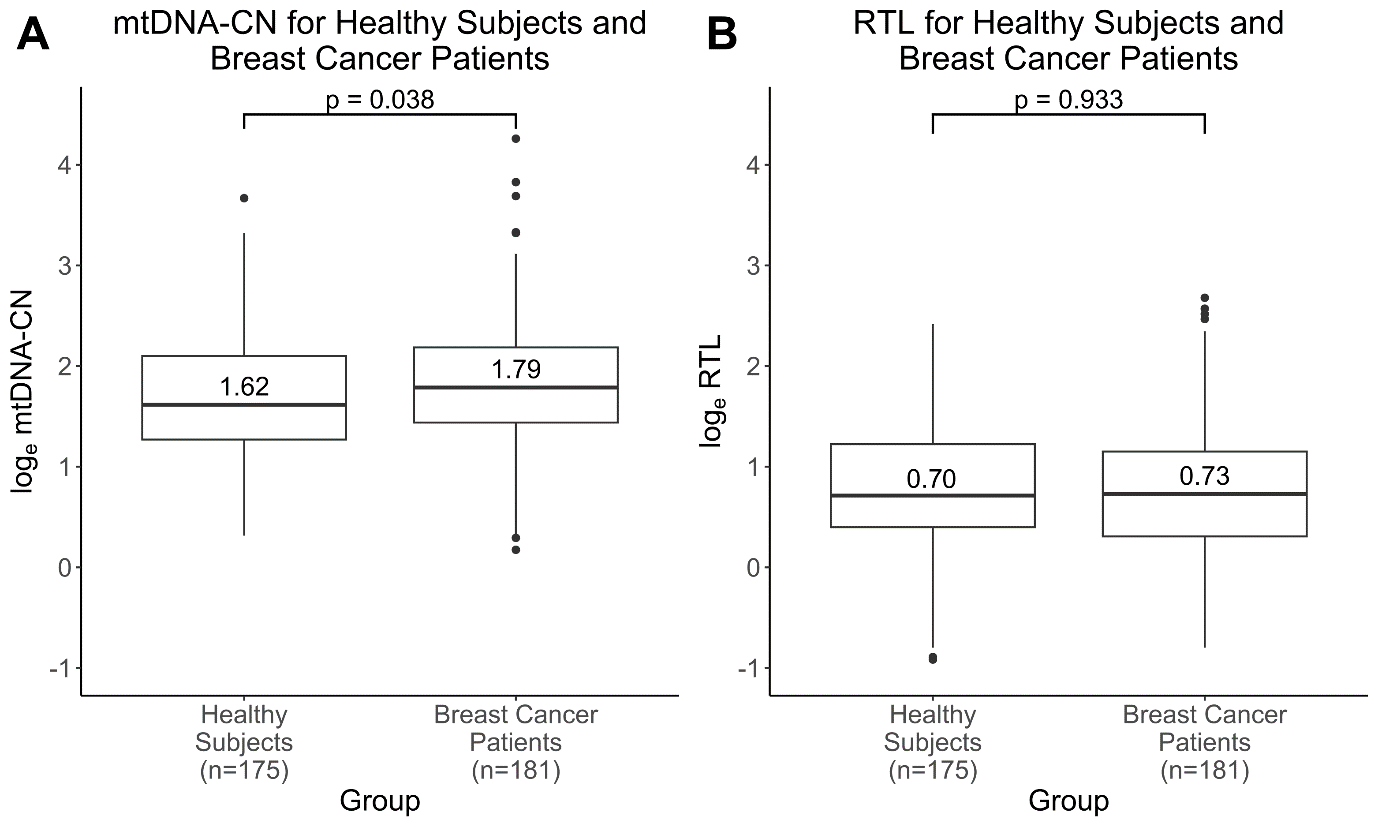
**

**Figure S2. Univariate comparison of peripheral blood mtDNA-CN and RTL between healthy subjects and breast cancer patients**

The mtDNA-CN (A) and RTL (B) were compared between healthy subjects and breast cancer patients. The *p* values were calculated using Wilcoxon-Mann Whitney U test with p<0.050 as significant value. Middle line, median; Box, interquartile (Q1-Q3); Whisker, minimum-maximum value.
