# Supplementary material for: Higher peripheral blood mitochondrial DNA copy number and relative telomere length in under 48 years Indonesian breast cancer patients
Source: BMC Res Notes. 2024 Apr 28;17:120. doi: 10.1186/s13104-024-06783-y (PMC11057172; doi:10.1186/s13104-024-06783-y)
Supplement: Supplementary file 1 — Additional file 1. Figure S1. Flow diagram of the healthy subjects and breast cancer (BC) patients' enrolment Table S1. Comparison of mtDNA-CN and RTL between extraction methods Table S2. List of primer pairs Table S3. Characteristics of study participants Figure S2. Univariate comparison of peripheral blood mtDNA-CN and RTL between healthy subjects and breast cancer patients Figure S3. Univariate comparison of peripheral blood mtDNA-CN and RTL between under and above 48 years subgroup in healthy subjects and breast cancer patients. [file 13104_2024_6783_MOESM1_ESM.zip › Additional file/rev-Supplementary Figure 3.docx]

**
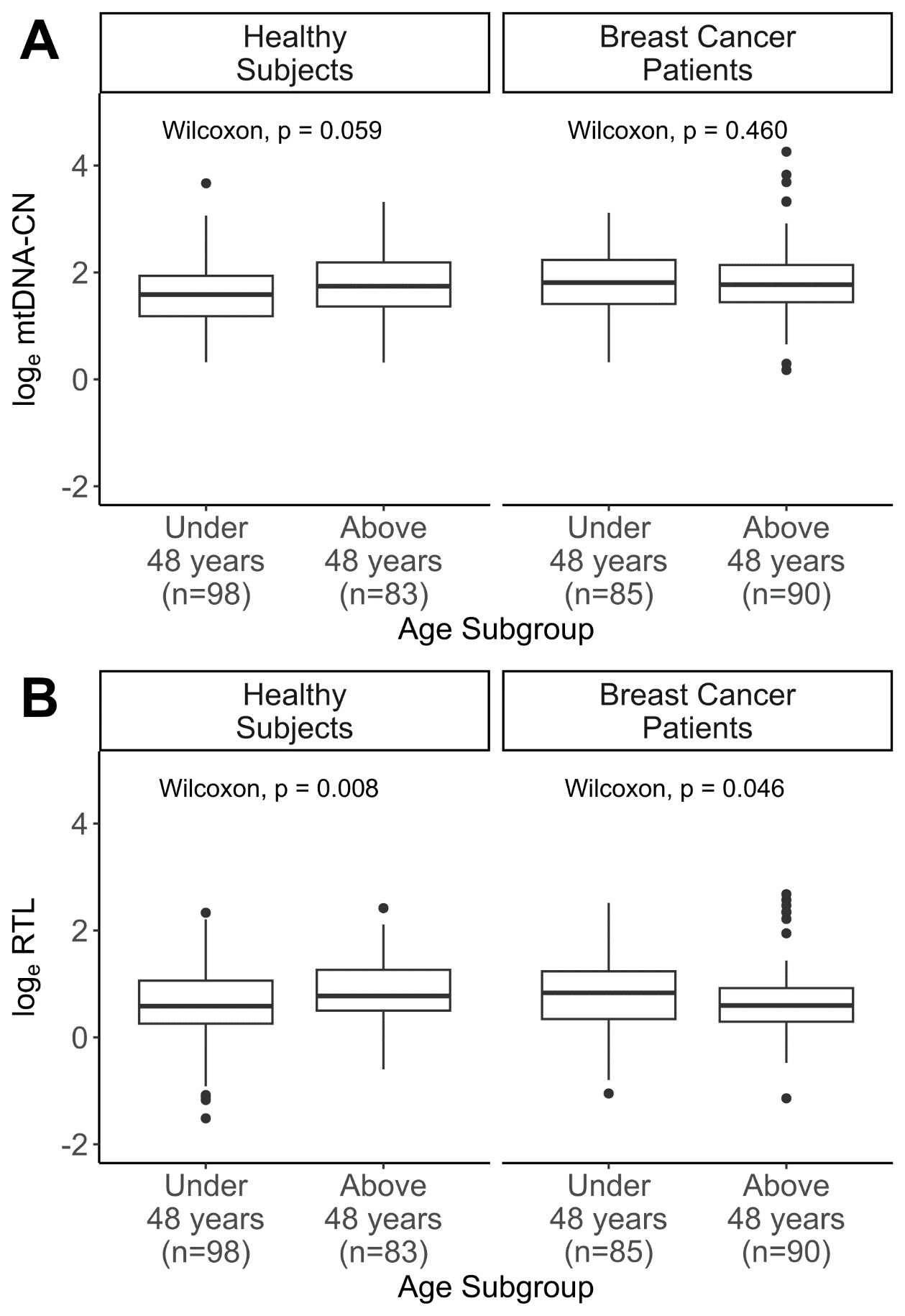
Figure S3. Univariate comparison of peripheral blood mtDNA-CN and RTL between under and above 48 years subgroup in healthy subjects and breast cancer patients**

The mtDNA-CN (A) was compared between under and above 48 years in healthy subjects (upper left) and breast cancer patients (upper right). The median values of mtDNA-CN for under and above 48 years in were 1.59 and 1.74 in healthy subjects, and 1.81 and 1.77 in breast cancer patients, respectively.

The RTL (B) was compared between under and above 48 years in healthy subjects (bottom left) and breast cancer patients (bottom right). The median values of mtDNA-CN for under and above 48 years in were 0.58 and 0.77 in healthy subjects, and 0.83 and 0.60 in breast cancer patients, respectively.

The *p* values were calculated using Wilcoxon-Mann Whitney U test with p<0.050 as significant value. Middle line, median; Box, interquartile (Q1-Q3); Whisker, minimum-maximum value.
