# Supplementary material for: Higher peripheral blood mitochondrial DNA copy number and relative telomere length in under 48 years Indonesian breast cancer patients
Source: BMC Res Notes. 2024 Apr 28;17:120. doi: 10.1186/s13104-024-06783-y (PMC11057172; doi:10.1186/s13104-024-06783-y)
Supplement: Supplementary file 1 — Additional file 1. Figure S1. Flow diagram of the healthy subjects and breast cancer (BC) patients' enrolment Table S1. Comparison of mtDNA-CN and RTL between extraction methods Table S2. List of primer pairs Table S3. Characteristics of study participants Figure S2. Univariate comparison of peripheral blood mtDNA-CN and RTL between healthy subjects and breast cancer patients Figure S3. Univariate comparison of peripheral blood mtDNA-CN and RTL between under and above 48 years subgroup in healthy subjects and breast cancer patients. [file 13104_2024_6783_MOESM1_ESM.zip › Additional file/rev-Supplementary Table 1.docx]

Table S1. Comparison of mtDNA-CN and RTL between extraction methods

| **Variable** | **QIAGEN Gentra Puregene Blood Kit** | **Geneius^TM^ Micro gDNA Extraction Kit** | ***p* value** |
| --- | --- | --- | --- |
|  | **n = 303** | **n = 53** |  |
| **MtDNA-CN** [median (IQR)] | 1.71 (1.30 - 2.16) | 1.81 (1.53 - 2.09) | 0.124 |
| **RTL** [median (IQR)] | 0.69 (0.29 - 1.22) | 0.71 (0.56 - 0.89) | 0.586 |

The *p* values were calculated using Wilcoxon-Mann Whitney U test.
