# Supplementary material for: Higher peripheral blood mitochondrial DNA copy number and relative telomere length in under 48 years Indonesian breast cancer patients
Source: BMC Res Notes. 2024 Apr 28;17:120. doi: 10.1186/s13104-024-06783-y (PMC11057172; doi:10.1186/s13104-024-06783-y)
Supplement: Supplementary file 1 — Additional file 1. Figure S1. Flow diagram of the healthy subjects and breast cancer (BC) patients' enrolment Table S1. Comparison of mtDNA-CN and RTL between extraction methods Table S2. List of primer pairs Table S3. Characteristics of study participants Figure S2. Univariate comparison of peripheral blood mtDNA-CN and RTL between healthy subjects and breast cancer patients Figure S3. Univariate comparison of peripheral blood mtDNA-CN and RTL between under and above 48 years subgroup in healthy subjects and breast cancer patients. [file 13104_2024_6783_MOESM1_ESM.zip › Additional file/rev-Supplementary Table 2.docx]

Table S2. List of primer pairs

| **Target Genes** | **Primer** | **Sequences (5’🡪3’)** | **Product Size (bp)** |
| --- | --- | --- | --- |
| *B2M* gene [24] | Forward (F594) | TGCTGTCTCCATGTTTGATGTATCT | 86 |
|  | Reverse (R679) | TCTCTGCTCCCCACCTCTAAGT |  |
| *MT-TL1* gene [24] | Forward (F3212) | CACCCAAGAACAGGGTTTGT | 107 |
|  | Reverse (R3319) | TGGCCATGGGTATGTTGTTA |  |
| Telomere repeats [25] | Forward (TELO-F) | CGGTTTGTTTGGGTTTGGGTTTGGGTTTGGGTTTGGGTT | 76 |
|  | Reverse (TELO-R) | GGCTTGCCTTACCCTTACCCTTACCCTTACCCTTACCCT |  |

Abbreviations: B2M, single-copy nuclear-encoded beta-2 microglobulin gene; MT-TL1, mitochondrially-encoded tRNA leucine 1 (UUA/G)
