# Supplementary material for: Higher peripheral blood mitochondrial DNA copy number and relative telomere length in under 48 years Indonesian breast cancer patients
Source: BMC Res Notes. 2024 Apr 28;17:120. doi: 10.1186/s13104-024-06783-y (PMC11057172; doi:10.1186/s13104-024-06783-y)
Supplement: Supplementary file 1 — Additional file 1. Figure S1. Flow diagram of the healthy subjects and breast cancer (BC) patients' enrolment Table S1. Comparison of mtDNA-CN and RTL between extraction methods Table S2. List of primer pairs Table S3. Characteristics of study participants Figure S2. Univariate comparison of peripheral blood mtDNA-CN and RTL between healthy subjects and breast cancer patients Figure S3. Univariate comparison of peripheral blood mtDNA-CN and RTL between under and above 48 years subgroup in healthy subjects and breast cancer patients. [file 13104_2024_6783_MOESM1_ESM.zip › Additional file/rev-Supplementary Table 3.docx]

Table S3. Characteristics of study participants

| **Variables** | **n** | **Healthy Subjects** | **Breast Cancer Patients** | ***p*** |
| --- | --- | --- | --- | --- |
| Age (years, median [IQR]) | 356 | 45.0 [38.0 - 53.0] | 48.0 [42.0 - 55.0] | **0.003** |
| BMI (kg/m^2^, median [IQR]) | 356 | 25.7 [23.5 - 29.3] | 24.8 [22.1 - 27.6] | **0.003** |
| TG (mg/dL, median [IQR]) | 356 | 99.0 [72.0 - 130.0] | 125.0 [90.5 - 174.0] | **<0.001** |
| HDL-C (mg/dL, median [IQR]) | 356 | 55.0 [49.0 - 64.0] | 49.0 [42.5 - 57.5] | **<0.001** |
| LDL-C (mg/dL, median [IQR]) | 356 | 139.0 [117.0 - 164.0] | 135.0 [112.0 - 165.0] | 0.588 |
| TC (mg/dL, median [IQR]) | 356 | 208.0 [182.0 - 238.0] | 202.0 [176.0 - 232.0] | 0.181 |
| FPG (mg/dL, median [IQR]) | 356 | 85.0 [80.0 - 91.0] | 88.0 [82.0 - 98.5] | **0.005** |
| TyG Index (median [IQR]) | 356 | 8.3 [8.0 - 8.7] | 8.6 [8.3 - 9.0] | **<0.001** |
| Alcohol consumption (n (%)) |  |  |  | **<0.001** |
| No | 327 | 178 (98.3%) | 149 (85.1%) |  |
| Yes | 29 | 3 (1.66%) | 26 (14.9%) |  |
| Smoking (n (%)) |  |  |  | **0.001** |
| No | 213 | 124 (68.5%) | 89 (50.9%) |  |
| Yes | 143 | 57 (31.5%) | 86 (49.1%) |  |
| Menarche age (n (%)) |  |  |  | 0.495 |
| < 12 | 29 | 17 (9.39%) | 21 (12.2%) |  |
| ≥ 12 | 315 | 164 (90.6%) | 151 (87.8%) |  |
| Menopause (n (%)) |  |  |  | 0.264 |
| No | 120 | 56 (51.4%) | 64 (43.5%) |  |
| Yes | 136 | 53 (48.6%) | 83 (56.5%) |  |
| Childbirth history (n (%)) |  |  |  | **<0.001** |
| No | 48 | 36 (22.4%) | 12 (7.50%) |  |
| Yes | 273 | 125 (77.6%) | 148 (92.5%) |  |
| Breastfeeding (n (%)) |  |  |  | **<0.001** |
| No | 68 | 48 (26.5%) | 20 (11.4%) |  |
| < 12 months | 87 | 31 (17.1%) | 56 (32.0%) |  |
| ≥ 12 months | 201 | 102 (56.4%) | 99 (56.6%) |  |
| Hormonal contraceptive use (n (%)) |  |  |  | **0.006** |
| No | 214 | 122 (67.4%) | 92 (52.6%) |  |
| Yes | 142 | 59 (32.6%) | 83 (47.4%) |  |

IQR, interquartile range; BMI, body mass index; TG, triglycerides; HDL-C, high-density lipoprotein cholesterol; LDL-C, low-density lipoprotein cholesterol; TC, total cholesterol; FPG, fasting plasma glucose; TyG, triglyceride and glucose.

The *p* values were calculated using Wilcoxon-Mann Whitney U test for continuous variables and Pearson’s chi-squared test for categorical variables. The significant *p* values are in bold (*p* <0.050).
